# Tropical South Atlantic influence on Northeastern Brazil precipitation and ITCZ displacement during the past 2300 years

**DOI:** 10.1038/s41598-018-38003-6

**Published:** 2019-02-08

**Authors:** Giselle Utida, Francisco W. Cruz, Johan Etourneau, Ioanna Bouloubassi, Enno Schefuß, Mathias Vuille, Valdir F. Novello, Luciana F. Prado, Abdelfettah Sifeddine, Vincent Klein, André Zular, João C. C. Viana, Bruno Turcq

**Affiliations:** 1 Geosciences Institute, University of São Paulo, Rua do Lago 562, 05508-080 São Paulo, Brazil; 2 Andaluz Institute of Earth Sciences, CSIC-University of Granada, Granada, Spain; 3 IRD-Sorbonne Universities (UPMC, Univ. Paris 06) - CNRS-MNHN, LOCEAN Laboratory, Center IRD France-Nord, F-93143 Bondy, France; 4 MARUM - Center for Marine Environmental Sciences, University of Bremen, D-28359 Bremen, Germany; 5 Department of Atmospheric and Environmental Sciences, University at Albany, Albany, NY 12222 USA; 6 Geosciences Institute, University of Brasília, Brasília, 70910-900 Brazil; 7 Federal University of Bahia, Instituto de Biologia, 40170-115 Salvador, Brazil; 8 Department of Geochesmistry, Fluminense Federal University, 24020-141 Niterói, Brazil

## Abstract

Recent paleoclimatic studies suggest that changes in the tropical rainbelt across the Atlantic Ocean during the past two millennia are linked to a latitudinal shift of the Intertropical Convergence Zone (ITCZ) driven by the Northern Hemisphere (NH) climate. However, little is known regarding other potential drivers that can affect tropical Atlantic rainfall, mainly due to the scarcity of adequate and high-resolution records. In this study, we fill this gap by reconstructing precipitation changes in Northeastern Brazil during the last 2,300 years from a high-resolution lake record of hydrogen isotope compositions of plant waxes. We find that regional precipitation along the coastal area of South America was not solely governed by north-south displacements of the ITCZ due to changes in NH climate, but also by the contraction and expansion of the tropical rainbelt due to variations in sea surface temperature and southeast trade winds in the tropical South Atlantic Basin.

## Introduction

Northeastern Brazil (NEB), also known as Nordeste, is one of the most vulnerable regions to climate change in South America. During the last decades, the NEB has experienced a drastic reduction in precipitation causing desertification expansion faster than anywhere else on the continent^1^. The causes of such anomalous climatic conditions remain elusive and probably are driven by several processes, which are still not fully understood. A better comprehension of these processes is of particular societal relevance since the NEB is densely populated and currently facing severe problems of water supply.

**Figure 1:**
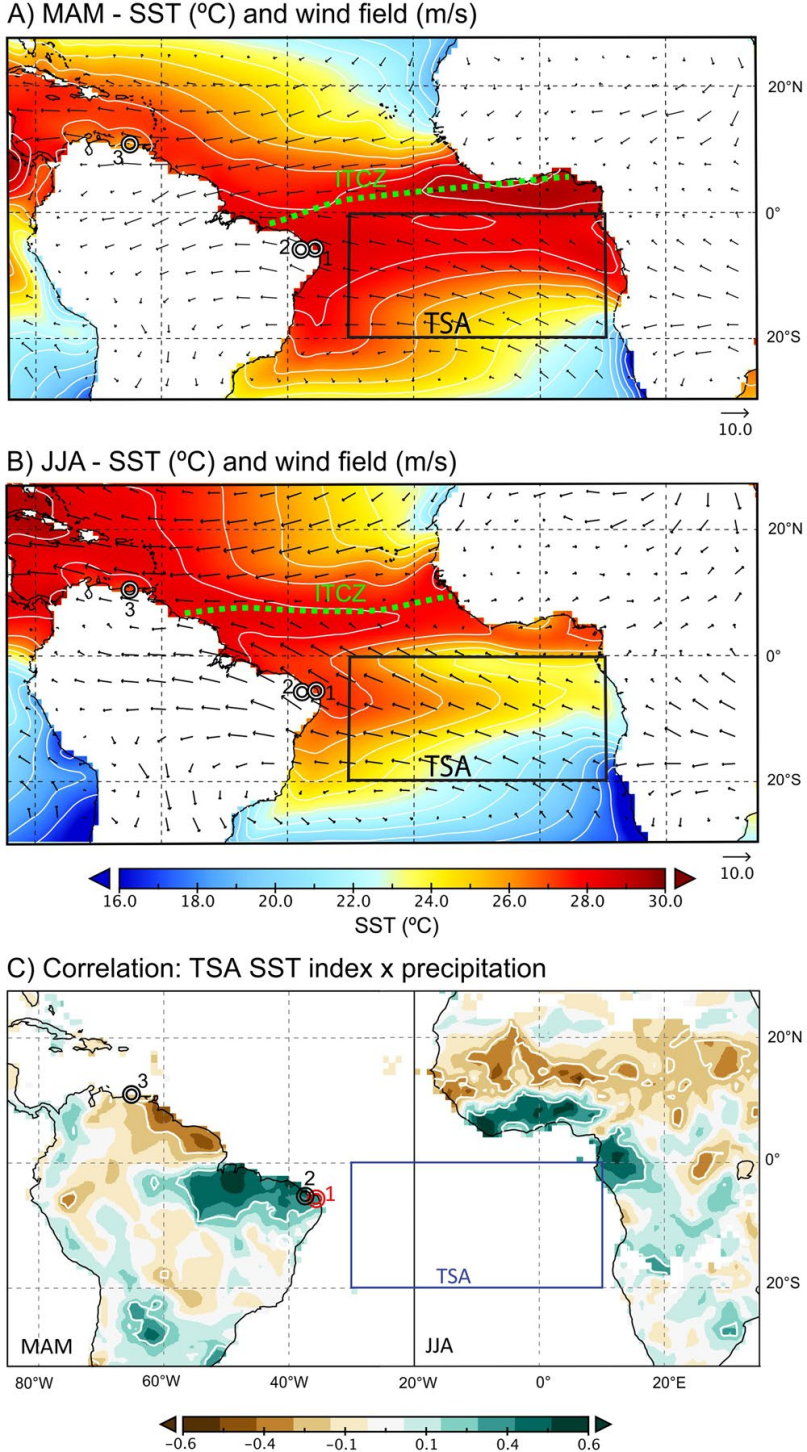
Tropical Atlantic climatology. Mean climatological sea surface temperature (SST) (°C) and 850 hPa wind field (m/s) over the tropical Atlantic Basin for (**A**) March, April and May (MAM) and (**B**) June, July and August (JJA). Both fields are averaged over the period 1982–2016. Contour interval for SST (white lines) is 1 °C. SST data are based on NOAA Optimum Interpolation SST V2^55^, and wind field is from ERA-Interim reanalysis data^56^. ITCZ plots are based on maximum mm.day^−1^ in MAM and JJA^57^. (**C**) Cross-equatorial and trans-hemispheric co-variance between precipitation in NEB Brazil and West Africa due to tropical South Atlantic SST variability represented by the correlation between Tropical South Atlantic (TSA) SST index (SST averaged over 0–20 S, 10E–30 W (blue box), calculated from HadISST and NOAA OI data sets^9,58^ with CRU TS3.24 precipitation data^59^. Correlations are for respective rainy seasons (MAM in South America and JJA in Africa) over period 1948–2014. White contour lines indicate regions with correlations significant at p < 0.05. Study sites: (1) Boqueirão Lake and Ceará-Mirim IAEA Station; (2) Fortaleza Station; (3) Cariaco Basin^14^.

Despite the dramatic decrease in precipitation over NEB as a whole, there are significant differences between the northern and eastern coastal sectors. Although both are influenced by the Tropical Atlantic Ocean, most of the NEB, especially the northern area, is primarily influenced by the seasonal displacement of the ITCZ reaching its southernmost position during austral autumn^2^ (March to May, MAM). The ITCZ is defined as a maximum in tropical precipitation^3^ or as a tropical belt of convective clouds – tropical rainbelt^4^, and its mean position varies seasonally from 9°N to 2°N over the Atlantic Ocean^5^. During the seasonal ITCZ shift to the south, higher precipitation in NEB is associated with warmer sea surface temperatures (SST) in the Tropical South Atlantic (TSA) and weaker Southeast (SE) trade winds^5,6^ (Fig. 1A). Indeed, tropical warming in the trade wind convergence zone promotes the ascent of warm and moist air, contributing to deep convective cloud formation. At higher levels, divergence leads to poleward flow and subsidence over subtropical latitudes, where near-surface flow is redirected towards the equator, closing the meridional Hadley cell^5^.

In contrast to austral autumn, the austral winter (June to August, JJA) is characterized by significant cooling in the TSA, stronger SE trade winds that cross the Equator and a northward displacement of the ITCZ^5^ (Fig. 1B). Additionally, in contrast to the northern region, precipitation over the eastern coastal NEB is modulated by the sea breeze circulation and easterly waves disturbances (EWD), which propagate westward over the tropical South Atlantic Ocean and are intensified by the SE trade winds during JJA^7,8^. This meridional gradient is also known as Atlantic Meridional Mode, where TSA and the tropical North Atlantic (TNA) can vary independently on decadal time scales (e.g.^9–13^). Other zonal oceanic modes in the tropics may also influence the climate in NEB, such as the Atlantic Equatorial Mode (AEM)^6,12,13^ or the El Niño Southern Oscillation (ENSO). In contrast to Meridional Mode, the AEM and ENSO records usually do not contain sufficient temporal resolution to allow a discussion of its possible influence. Moreover, the AEM amplitude and impact on an interannual basis are relatively small and hardly detectable in decadal to centennial paleoclimate records.

Northward ITCZ displacements in response to warm NH temperature anomalies during the Holocene are by far the most commonly invoked cause explaining climate variability across the tropical Atlantic coastal regions such as NEB or the Cariaco Basin^14–17^. However, these previous studies may have overlooked other potential climatic mechanisms related to Equatorial Atlantic SST variations, owing to the lack of robust high-resolution rainfall and SST proxy records in this area, thus preventing an accurate assessment of changes in precipitation due to SST gradients and hemispheric temperature asymmetries, especially during the last two millennia. High positive correlation between modern TSA-SST and precipitation over the Tropical Atlantic suggests that precipitation over NEB could also be related to TSA-SST variability (Fig. 1C). Furthermore, the Northern Hemisphere-Southern Hemisphere stack reconstruction of tropical precipitation for the last millennia^17^ only includes records from sites within the South American Monsoon domain, due to a complete lack of ITCZ precipitation records south of the equator along coastal South America.

Here we address this issue by investigating changes in precipitation in the NEB using lacustrine sediments collected from Boqueirão Lake (5°14′S; 35°32′W) spanning the last 2,300 years (yrs). Our study is based on a high-resolution record of the hydrogen isotope composition of the *n*-C_28_ alkanoic acid (δD_wax_) from the core Boqc0901 dated by Viana *et al*.^16^ with an average of 13 years resolution. Changes in δD_wax_ reflect changes in δD_precip_ used by terrestrial plants for biosynthesis of wax lipids^18,19^. As secondary factors, vegetation type and relative humidity changes can also affect δD_wax_^20^. To minimize such biases, we corrected the isotopic composition of δD_wax_ for vegetation type changes (see Methods).

Boqueirão Lake is a small lake formed by dune blockage with a limited catchment area in coastal sand dune fields under a semi-arid climate in NEB^21^. The adjacent sandy soils to this lake are covered predominantly by a dry forest known as “caatinga”. These environmental conditions result in poor soil production with low organic matter content, which reduces the retention time of the lipids in soils before discharge and deposition, contrary to large river catchments^22,23^. Boqueirão Lake is located near the eastern coast, where ~50% of precipitation originates from the seasonal ITCZ overpass during MAM, ~40% is brought by SE trade winds and sea breezes during JJA, and the remaining 10% distributed throughout the other months^5,24^ (Fig. 1A,B).The spatial correlation fields between δ^18^O and ITCZ-driven MAM precipitation obtained from two meteorological stations at Fortaleza and Ceará-Mirim (International Atomic Energy Agency - Global Network of Isotopes in Precipitation -IAEA-GNIP - stations) indicate that isotopic values in rainfall are decreasing when the precipitation amount increases (Fig. S1). This negative correlation highlights the link between the stable oxygen and, consequently, also the hydrogen isotope ratios with rainfall amount during the southernmost ITCZ position in MAM. Furthermore, δD and δ^18^O values of precipitation fall on the global meteoric water line (GMWL) (Fig. S2). Overall these results indicate that the isotopic composition of rainfall in our study region is dominated by the amount effect^25^.

Differences in rainfall regimes between these two GNIP stations occur during JJA, when the SE trades are intensified and the ITCZ is displaced northward, leading to a strong decrease in precipitation (higher δ^18^O and δD values) at Fortaleza. On the other hand, precipitation remains high at Ceará-Mirim, due to sea breezes and EWDs affecting the eastern part of Northeastern Brazil during the winter (JJA) rainy season^26–28^, similar to what occurs over nearby Boqueirão Lake. Although the amount effect is still significant in both Fortaleza and Ceará-Mirim GNIP stations, the correlation is significantly lower in Ceará-Mirim (R^2^ = 0.61) than in Fortaleza (R^2^ = 0.81) due to the influence of the more enriched winter rainfall (Fig. S3). Despite these regional differences in rainfall regime, more negative δD and δ^18^O values at both stations are only associated with the intensification of ITCZ precipitation in NEB. This relationship is supported by the significant negative spatial correlation between the δ^18^O of precipitation at the two stations and rainfall amount throughout the study region during MAM (Fig. S1). Furthermore, the vegetative growth in the caatinga vegetation of semi-arid Northeastern Brazil occurs mainly during the beginning of the rainy season^29–31^. The trees in the caatinga respond with immediate leaf growth to the first rainfall events associated with the ITCZ regime after the long dry season from March to May. Assuming that little or no wax is produced after the leaves become fully developed^20,32,33^, similarly to the semi-arid setting in the African Sahel^18^, the hydrogen isotopic composition of the leaf waxes is primarily associated with the isotopic signature of precipitation related to the early ITCZ regime in NEB.

The environmental characteristics of Boqueirão Lake make it an ideal location to scrutinize past climatic variability and related forcings along the tropical Atlantic shore. We compare our record with the Ti record from Cariaco Basin to assess the coherence and synchronicity of precipitation changes along the northeastern South American coast.

## Results and Discussion

**Figure 2:**
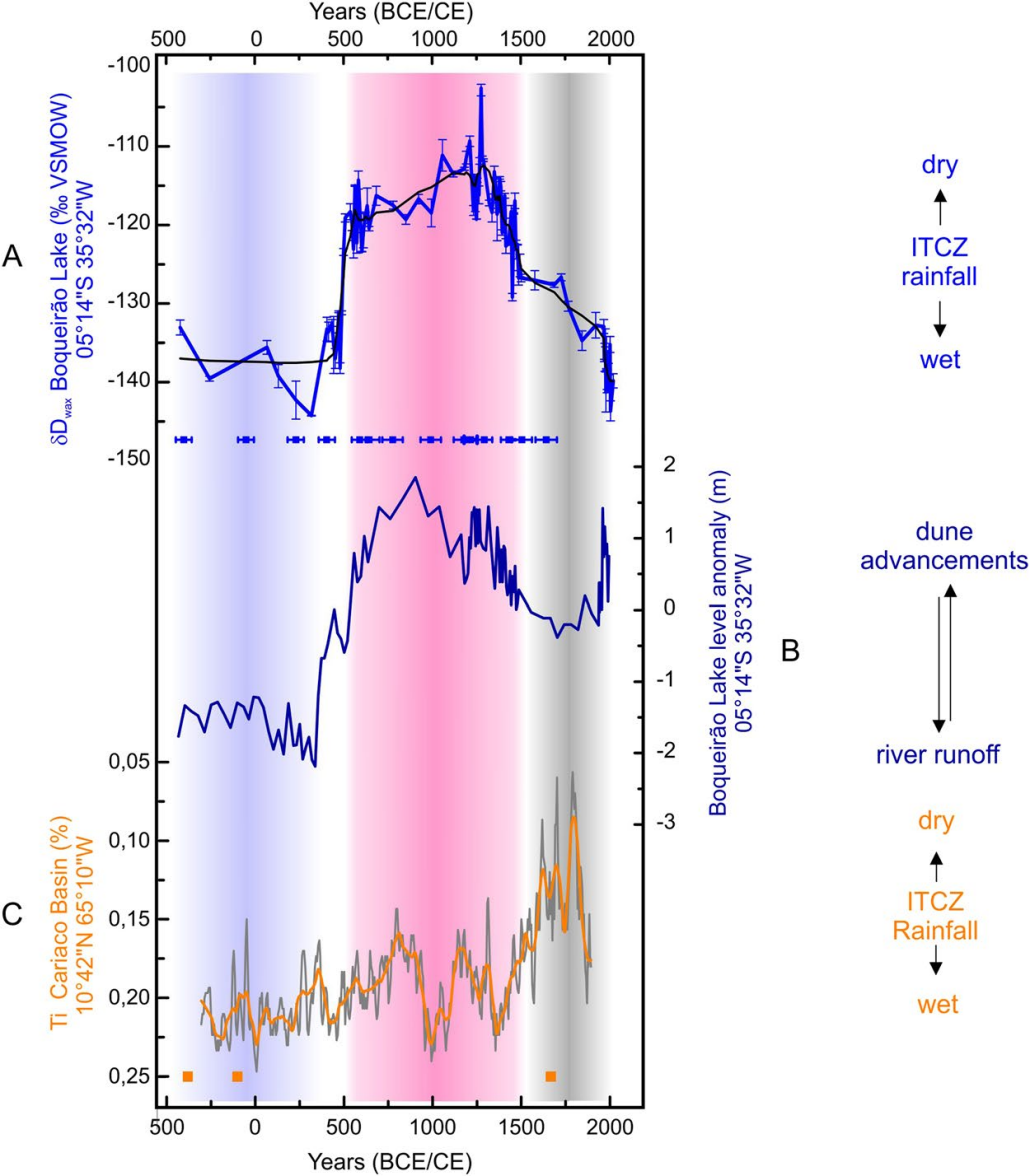
Record comparison between (**A**) δD_wax_ record of *n*-C_28_ alkanoic acid from Boqueirão Lake sediment core Boqc0901 in Northeastern Brazil (NEB), black line represents a smoothing with a 20-point window (this study) with ^14^C AMS ages identified by blue symbols^16^; (**B**) Boqueirão Lake level reconstruction^16^; (**C**) Ti record from Cariaco Basin, Venezuela^14^, orange line represents a smoothing with a 20-point window and orange symbols ^14^C AMS ages^14^. All smoothed records were calculated according to Savitzky-Golay method^60^ performed by the software Origin 8.0. Blue shaded area from 420 BCE to 500 yrs CE indicates a wet period (in-phase) at Boqueirão lake and Cariaco Basin, red shaded area from 500 to 1500 yrs CE indicates a dry period in Boqueirão Lake and wet in Cariaco (antiphased) and gray shaded area from 1500 to 1830 yrs CE indicates wet conditions in Boqueirão and dry in Cariaco (antiphased). The running-mean correlation results are presented in the Supplementary Material.

Our record documents a predominantly humid period between 500 yrs BCE and 420 yrs CE associated with low δD_wax_ values ranging from −130 to −145‰. These climate conditions are followed by an abrupt aridification and a long dry phase from 500 to 1,300 yrs CE as revealed by higher δD_wax_ values reaching −100‰ (Fig. 2A). Between 1580 and 1900 yrs CE, the δD_wax_ values rapidly declined, reaching values similar to those prior to 420 yrs CE, thus characterizing a long humid period during the Little Ice Age (LIA) in NEB.

There is a striking resemblance between our δD_wax_ record and the reconstructed Boqueirão Lake level variations for the last 2,000 yrs based on a diatom transfer function in the same sediment core Boqc09/01 (Fig. 2A,B). However, the periods with marked increase (decrease) of ITCZ-related precipitation indicated by lower (higher) δD_wax_ values occur unexpectedly during periods of significant lake level drop (rise) reconstructed by Viana *et al*.^16^ and Zocatelli *et al*.^34^. This inconsistency can be explained by the strong influence of aeolian processes on the Boqueirão Lake level^21^ which was not considered in previous studies. Boqueirão is a coastal lake that originated from sand-dune damming of a small freshwater river (Fig. S4). Our isotopic data suggest that the lake level high-stands are associated with advancing dunes over the drainage that led to damming during dry periods with reduced river flow. Conversely, during wetter climate periods the increased drainage flow would most likely erode the dune dam and pass through any obstruction, resulting in a lowering of the lake water level.

To investigate latitudinal ITCZ displacements, we compare the data from Boqueirão Lake to the Ti record from Cariaco Basin^14^ (Fig. 2C), which is one of the most commonly used proxies for past ITCZ locations over the tropical North Atlantic. Because our lake record is located close to the southern limit of the ITCZ domain in South America, we evaluated expansion and contraction of the tropical rain belt by comparing our record with the Cariaco Basin, which is located close to the northern limit of the ITCZ.

In the earliest part of our record (i.e. before ~500 yrs CE), relatively humid conditions prevail in both NEB and Cariaco (correlation <−0.5, p < 0.05, see Supplementary Material), which is not consistent with the traditional pattern of meridional ITCZ displacement regulating precipitation at both sites (Fig. 2). Our new results, combined with the Cariaco record, instead suggest an expansion of the tropical rainbelt that would correspond to a longer rainy season in both hemispheres. This in-phase relationship might be a consequence of a general warming of the Atlantic Equatorial Basin. However, this hypothesis would need to be further supported by new and high-resolution SST reconstructions from the tropical Atlantic for the last millennia.

On average for the whole tropical Atlantic, a warming of the sea surface temperature was documented over the last decades associated with strengthened trade winds without significant long-term changes in the mean position of the ITCZ, although the maximum northern and southern ITCZ displacements occurred during these decades^13^. Alternatively, other mechanisms could also influence the width of the tropical rainbelt, such as cloud-radiative feedbacks^35^, or variations in the tropical moist-static energy budget^36^. Our record is also in agreement with evidence of a tropical rainbelt expansion before ~250 yrs CE from the Pacific ITCZ domain^37^.

NEB faced a very long dry period between 500 and 1,500 yrs CE, which is consistent with a relatively far northern mean position of the ITCZ (Fig. 2A). This climate scenario is consistent with the overall wet conditions in Cariaco relative to the large dry event that characterizes the Little Ice Age (LIA). Humid conditions in Cariaco peaked around 1,000 yrs CE, corresponding to the Medieval Climate Anomaly^14^, and were bounded by two dry periods lasting from approximately 600 to 800 yrs CE and from 1,100 to 1300 yrs CE (Fig. 2C).

We associate the northward displacement of the ITCZ with the reinforcement of the SE trade winds, resulting in a shallower equatorial thermocline and TSA cooling^12^. This would promote a long dry phase in NEB (Fig. 2A), as expected for a northward displacement of the ITCZ^12^. We therefore conclude that the large shift in precipitation at ~500 yrs CE in the NEB was strongly influenced by temperature changes in TSA and intensified SE trade winds, as an important, and hitherto overlooked, contributing factor working in conjunction with warmer NH temperature.

Around 1500 yrs CE, NEB climate abruptly transitioned towards wetter conditions, as a consequence of a more southerly position of the ITCZ, affecting latitudes as far south as 5°S. This is consistent with a dry climate recorded in Cariaco from 1600 to 1850 yrs CE during the LIA^14^ (Fig. 2, see also S9 in Supplementary Material) and also with the ITCZ stack reconstruction from Lechleitner *et al*.^17^. This period of ITCZ rainfall extending into the southern hemisphere tropics is commonly associated with cooler temperatures in the northern hemisphere^38–40^. Indeed, a modern-day record of colder TNA and warmer TSA confirms a southern displacement of the ITCZ and stronger precipitation over NEB^40^.

During the LIA, Denniston *et al*.^37^ suggested a contraction of the Indo-Pacific tropical rain belt, however, given the lack of proxy records in the Atlantic margin, it is not possible to accurately constrain the contraction of the ITCZ over the tropical Atlantic domain.

It is well known that ENSO is associated with rainfall variability in NEB^5,41^. Indeed, there is a delayed anomalous warming of the tropical North Atlantic during El Niño events, which reduces northeast trade winds and favors the ITCZ displacement to the north, while the opposite mechanism occurs during La Niña events^42,43^. The past variability of ENSO during the last 2 millennia is still not clearly understood due to large inconsistencies among the existing reconstructions (Fig. S5). Such major differences might be attributed to regional features, dating uncertainties, distinct response to ENSO or a non-linear behavior of the proxy-climate relationship (e.g.^44–52^). When comparing the ENSO records from the eastern Pacific with our NEB isotope data over the last 2,300 years, we do not find a clear evidence for an in-phase relationship as expected from the modern climatology (Fig. S5). In addition, the anticipated in phase relationship of long-term precipitation in Nordeste and western Pacific ENSO records or indexes is neither so apparent (Fig. S5D,E).

Our lake record suggests that the ITCZ activity, as represented by the so-called ‘meridional shift of the tropical rain belt’ hypothesis^14,17^ needs to be discussed with caution because it cannot fully explain the long-term precipitation variability over NEB during the last 2,300 years. We show here that the NH climate is not the sole driver of NEB precipitation and the tropical Atlantic needs to be considered as an additional important factor influencing the tropical rainbelt dynamics.

## Methods

### Lipid extraction, quantification and identification

Samples were prepared at LOCEAN-UPMC (Laboratoire d’Océanographie et du Climat, Expérimentations et Approches Numériques – Université Pierre et Marie Curie, Paris). Lipids from 89 sediment samples of the Boqc09/01 core were extracted by ultrasonication with a DCM/MeOH (3:1) solvent mixture. After saponification of the resulting total lipid extracts with 4 M KOH/MeOH, removal of the neutral fraction with hexane and acidification of the residue, the acid fraction was recovered with hexane/ethyl acetate (9:1), methylated using BF3 and further purified by column chromatography over silica gel and elution with DCM:hexane (2:1). Fatty acid methyl esters (FAME) were analyzed on Agilent 6890 N gas chromatograph (GC) using flame ionization detection (FID). Quantification of compounds was performed by peak area integration in FID chromatograms relative to the internal standard 5β-cholenic acid added prior to extraction. In order to confirm compound identification selected samples were analyzed by GC-MS on an Agilent 7890 GC coupled to an Agilent 5975 mass spectrometer detector. High values of Carbon Preservation Index (CPI) obtained, between 7.7 and 11.1, indicate good plant wax preservation. Concentrations of individual long-chain fatty acids (C_24–32_) range between 20 and 180 µg/g in the dry sediment samples. Taking into account that the pattern of long-chain fatty acids were very similar to other fatty acids, for instance *n*-C_30_ alkanoic acid, we chose to analyze *n*-C_28_ alkanoic acids, which is very close to the average carbon length in our samples (27.4) and has major quantities per gram (C-28 average 7.2 mg/g; C-30 average 5 mg/g), resulting in higher confidence, reliability and reproducibility of our results.

### Isotope analysis

Compound-specific isotope analyses of *n*-C_28_ alkanoic acids were performed at MARUM - Center for Marine Environmental Sciences, Bremen, Germany. Compound-specific δD analyses were performed on a Thermo Trace GC equipped with an Agilent DB-5 column (30 m × 0.25 mm × 0.25 μm) coupled to a Thermo Fisher Scientific MAT 253 IRM-MS via a pyrolysis interface operated at 1420 °C. Measurements were calibrated against ^2^H reference gas with known isotopic composition and the H^3+^ factor was monitored daily (values varied between 6.7 and 6.9). δD values are reported in permil (‰) relative to Vienna Standard Mean Ocean Water (VSMOW). An external standard mixture with known δD values was analyzed repeatedly every six runs, yielding a long-term mean standard deviation of <3‰ and a mean deviation of <1‰ from reference values. Stable carbon isotope compositions (δ^13^C) of the same compounds were measured using the same type of GC and GC column coupled to a Finnigan MAT 252 IRM-MS via a modified combustion interface at 1000 °C. Calibration of carbon isotopes was achieved by comparison to CO_2_ reference gas. δ^13^C values are reported in permil (‰) against Vienna Pee Dee Belemnite (VPDB). An external standard mixture was analyzed repeatedly every 6 runs and yielded a long-term mean standard deviation of 0.2‰ with a mean deviation of 0.1‰ from the reference values. Samples were measured in duplicates for δD and δ^13^C. Mass balance calculations were made for removal of the isotopic contribution of the added methyl group. Mean propagated errors are 3‰ for δD and 0.2‰ for δ^13^C. Isotopic results obtained from *n-*C_28_ alkanoic acids of Boqueirão Lake core Boq09/01 are reported as δD_wax_ and δ^13^C_wax_.

### Correction of δD_wax_ for vegetation-type

To remove fractionation effects based on vegetation type changes, we corrected the isotopic composition of δD_wax_ based on the δ^13^C_wax_ signal. The δ^13^C_wax_ varied from −26.1‰ to −32.5‰ (±0.5‰) (Fig. S6) indicating a predominance of the C3 plant signal during the last 2300 years. We tested a mixing model using the epsilon value weighted by different proportions of C3 and C4 vegetation, applying end-members of −35‰ and −22‰ for C3 and C4 plants (Fig. S6), respectively. To correct the δD_wax_, we weighted fractionation factor according to C3/C4 plants using fractionation factor (ε) of *n*-alkanes of C3 plants in tropical dry forests^53^ (ε = −125‰) and of C4 grasses in semi-arid environments^54^ (ε = −140‰), where the amount of precipitation is the most similar to NEB. Both end-members and fractionation factor were obtained according to compilation of Sachse *et al*.^20^.

The estimated precipitation (δD_precip-wax_) values were calculated according to the following equation: δD_precip-wax_ = [(δD_wax_ + 1000)/((ε/1000) + 1)] − 1000^20^. The results vary between 32‰ and −17‰ for ε weighted by C3/C4 (Fig. S7).

The results using fractionation factors weighted by C3/C4 from *n*-alkanes are consistent with the modern observed δD_precip_ (Fig. S8). However, there are no significant changes in δD_precip-wax_ patterns (Fig. S7). Therefore, we infer a weak influence of plant physiology and evapotranspiration on isotopic enrichment in soil and leaf waters in highly seasonal environments^18^ but discuss our results in terms of trends rather than absolute values.

## Supplementary information

Supplementary Material

## Data Availability

The dataset generated during the current study will be available in the PANGAEA.
