# Supplementary material for: Tropical South Atlantic influence on Northeastern Brazil precipitation and ITCZ displacement during the past 2300 years
Source: Sci Rep. 2019 Feb 8;9:1698. doi: 10.1038/s41598-018-38003-6 (PMC6368536; doi:10.1038/s41598-018-38003-6)
Supplement: Supplementary file 1 — Supplementary Material [file 41598_2018_38003_MOESM1_ESM.pdf]

# Tropical South Atlantic influence on Northeastern Brazil precipitation and ITCZ displacement during the past 2300 years

Giselle Utida*[1], Francisco W. Cruz[1], Johan Etourneau[2], Ioanna Bouloubassi[3], Enno Schefuß[4], Mathias Vuille[5], Valdir F. Novello[1], Luciana F. Prado[6], Abdelfettah Sifeddine[3,7], Vincent Klein[3], André Zular[1], João C.C. Viana[7], Bruno Turcq[3,8].

[1]Geosciences Institute, University of São Paulo, Rua do Lago 562, 05508-080, São Paulo, Brazil.

[2]Andaluz Institute of Earth Sciences, CSIC-University of Granada, Granada, Spain

[3]IRD-Sorbonne Universities (UPMC, Univ. Paris 06) - CNRS-MNHN, LOCEAN Laboratory, Center IRD France-Nord, F-93143 Bondy, France.

[4]MARUM - Center for Marine Environmental Sciences, University of Bremen, D-28359 Bremen, Germany.

[5]Department of Atmospheric and Environmental Sciences, University at Albany, Albany, NY 12222, USA.

[6]Geosciences Institute, University of Brasília, Brasília, 70910-900, Brazil

[7]Federal University of Bahia, Instituto de Biologia, 40170-115, Salvador, Brazil.

[8]Department of Geochesmistry, Fluminense Federal University, 24020-141, Niterói, Brazil.

*corresponding author (giselleutida@hotmail.com)

**SUPPLEMENTARY INFORMATION**

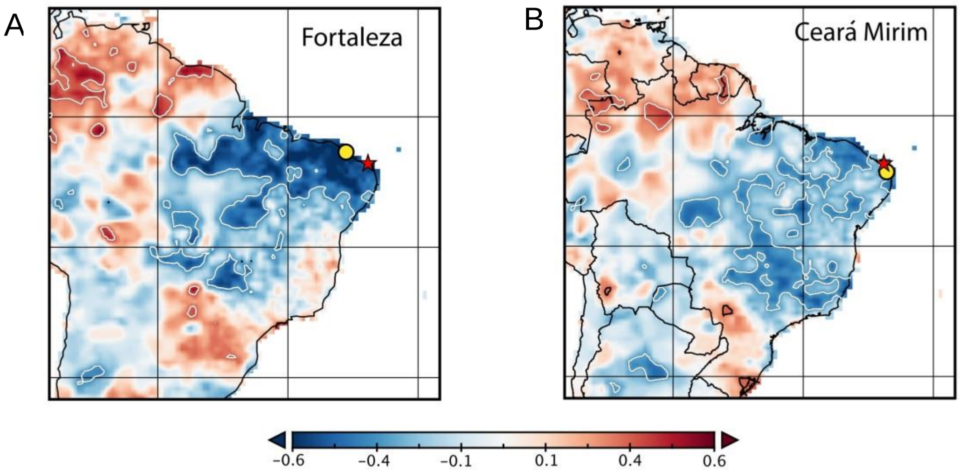


Figure S1 – Spatial correlation between MAM $\delta^{18}O$ at IAEA stations (A) Fortaleza and (B) Ceará-Mirim (yellow dots) and MAM precipitation[1] (1968-1985; data for 1977-1982 is missing). The white contours delimit correlations significant at $p<0.05$. The inland Fortaleza and coastal Ceará-Mirim stations are located 370 km and 45 km from Boqueirão Lake, respectively. The red star shows the location of Boqueirão Lake.

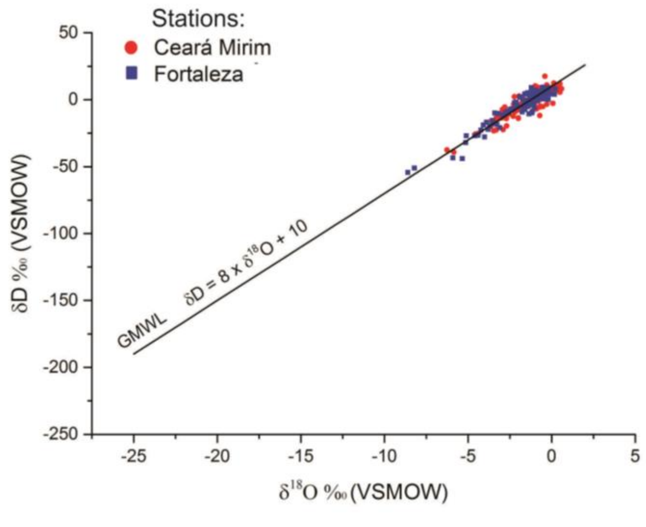


Figure S2 – Precipitation data of Fortaleza and Ceará-Mirim IAEA stations, $\delta D$ and $\delta^{18}O$ values of precipitation and the global meteoric water line (GMWL). GNIP-IAEA data was obtained from 1965 to 1985, and from 1961 and 1983, for Fortaleza and Ceará-Mirim stations, respectively.

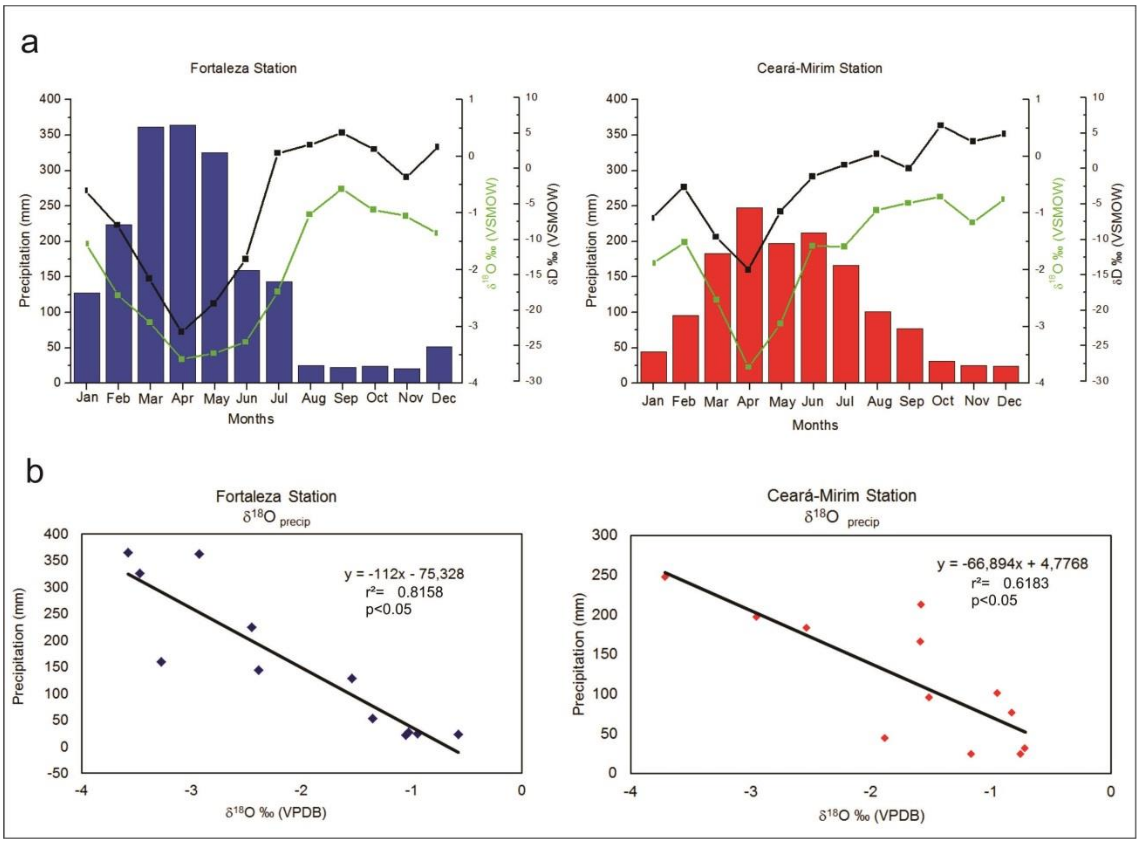


Figure S3 – a) Monthly precipitation amount and oxygen and deuterium isotopes registered at Fortaleza and Ceará-Mirim stations. b) Monthly oxygen isotope and precipitation amount correlation for Fortaleza and Ceará-Mirim stations. GNIP-IAEA data was obtained from 1965 to 1985, and from 1961 and 1983, for Fortaleza and Ceará-Mirim stations, respectively.

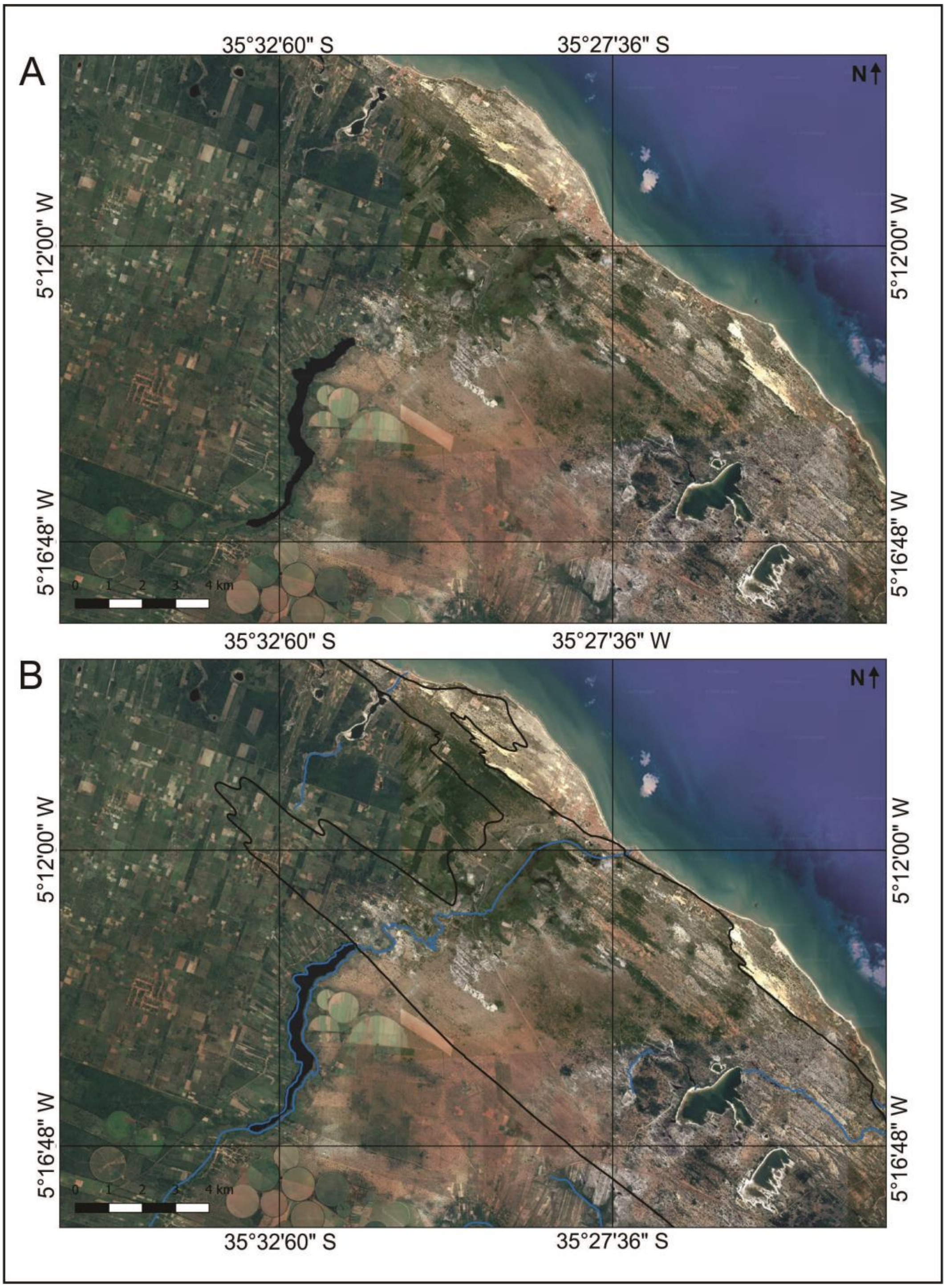


Figure S4. Study site. A) The Boqueirão Lake regional setting. B) Blue lines represent Boqueirão Lake and local drainage pathways whereas black lines depict dune advancements responsible for the formation of Boqueirão Lake during mid-Holocene (Zular et al., 2018). Map data: Google, Digital Globe 2018 image (www.earth.google.com[October 29, 2018]) set up using QGIS software version 3.4.0 (https://qgis.org/en/site/).

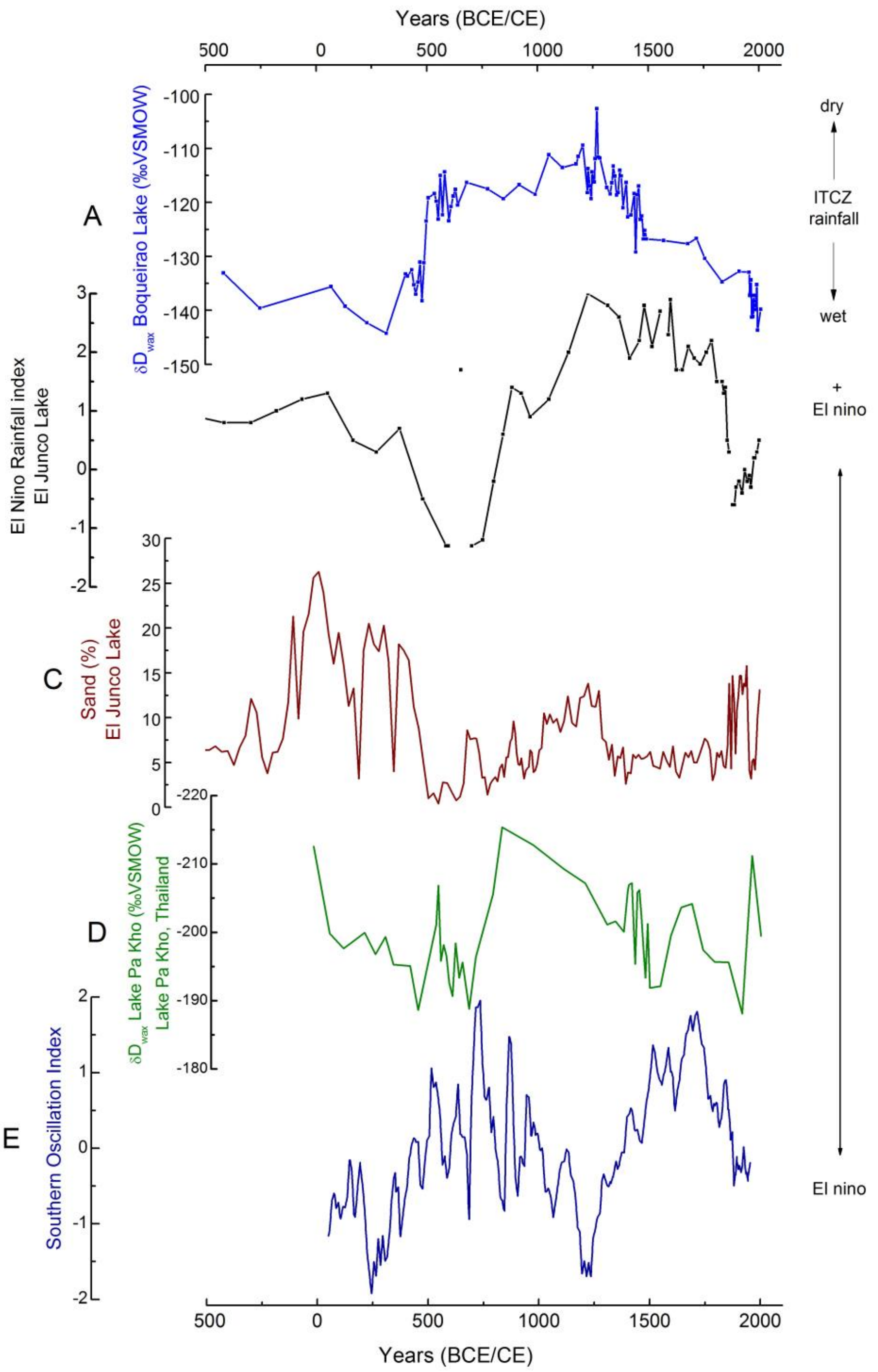


Figure S5 – Comparison between A) our $\delta D_{wax}$ record from the Boqueirão Lake sediment core Boqc0901 and the ENSO reconstructions: B) El Niño rainfall index[2,] and C) sand percentage[3] of El Junco Lake, Galapagos, D) $\delta D_{wax}$ of Lake Pa Kho, Northeastern Thailand[4], E) Southern Oscillation Index based on hydrological records of Pacific[5].

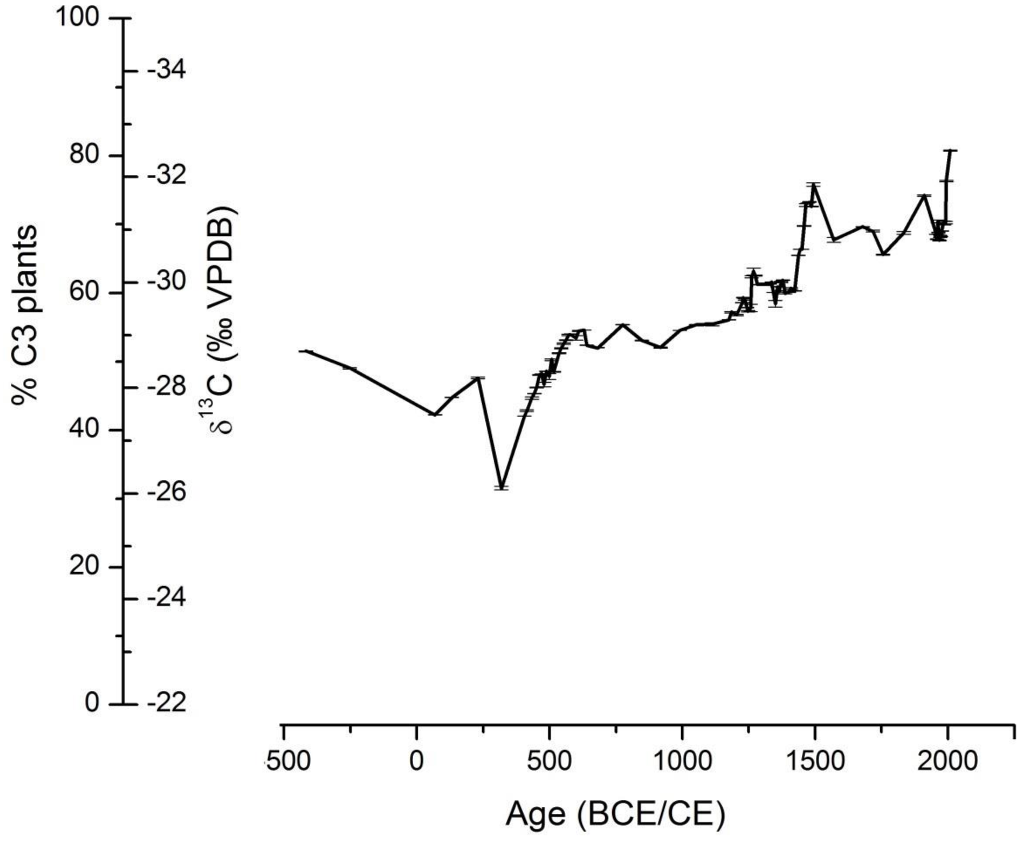


Figure S6 – Carbon isotope record of *n*-$C_{28}$ alkanoic acid and percentage of C3 plants in the Boqueirão Lake sediments. Percentage of C3 plants was added considering end-members as -35‰ and -22‰ for C3 and C4 plants, respectively, according to the most depleted $\delta^{13}C$ results of C3 and C4 plants[6].

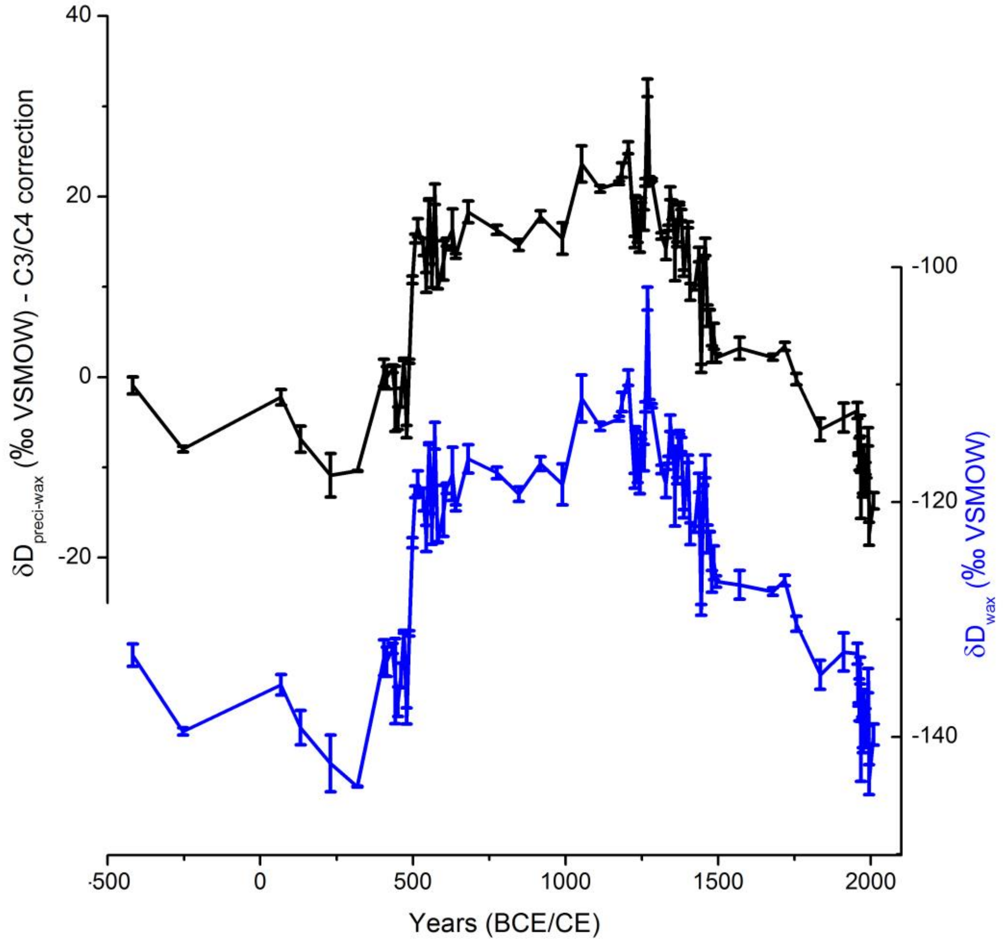


Figure S7 – $\delta D_{wax}$ obtained from the Boqueirão Lake core (blue line). $\delta D_{precip\text{-}wax}$ reconstructed considering C3 and C4 fractions (black line).

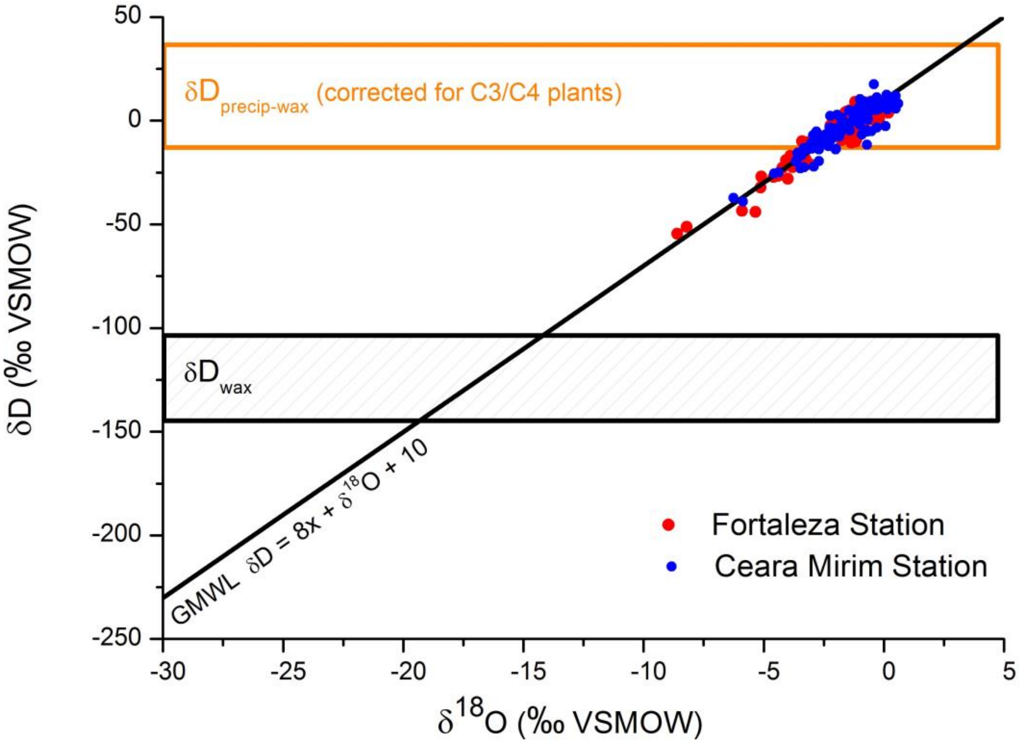


Figure S8 – $\delta^{18}O$ versus $\delta D$ of precipitation from Fortaleza and Ceará-Mirim GNIP-IAEA monitoring stations represented by red and blue dots, respectively, and Global Meteoric Water Line. Cross-hatched rectangle indicates the range of $\delta D_{wax}$ obtained from the Boqueirão Lake core and orange rectangle indicates the range of $\delta D_{precip\text{-}wax}$ reconstructed using $\varepsilon$ weighted by C3/C4 plants.

**Running mean-correlation**

The running-mean correlation ($r_m$)[7] was made using the software Past by applying a 20-year window of interpolated data from both the Boqueirão Lake $\delta D_{wax}$ and Cariaco Ti record[9] (Fig. S9). The Boqueirão and Cariaco records show a negative (positive) correlation when their climates are in-phase (anti-phased).

The running-mean correlation analysis between the Cariaco and Boqueirão records shows an in-phase relationship at the beginning of the records, which is steadily trending toward a more anti-phased behavior toward the present, according to the strong negative correlations, <-0.5 around 250 yrs BCE and positive correlations, >0.5 ($p<0.05$), around 1750 yrs CE. The correlation is less significant ($-0.4<r_m>0.4$, $p>0.05$) from ~400 CE to ~1,630 yrs CE. These results support our interpretation of humid conditions in both the Cariaco and Boqueirão records before 500 yrs CE. Between 500

and 1.500 yrs CE, there is a steady trend toward predominantly more anti-phased conditions, with highest correlation coefficients around 1,750 yrs CE, during the Little Ice Age.

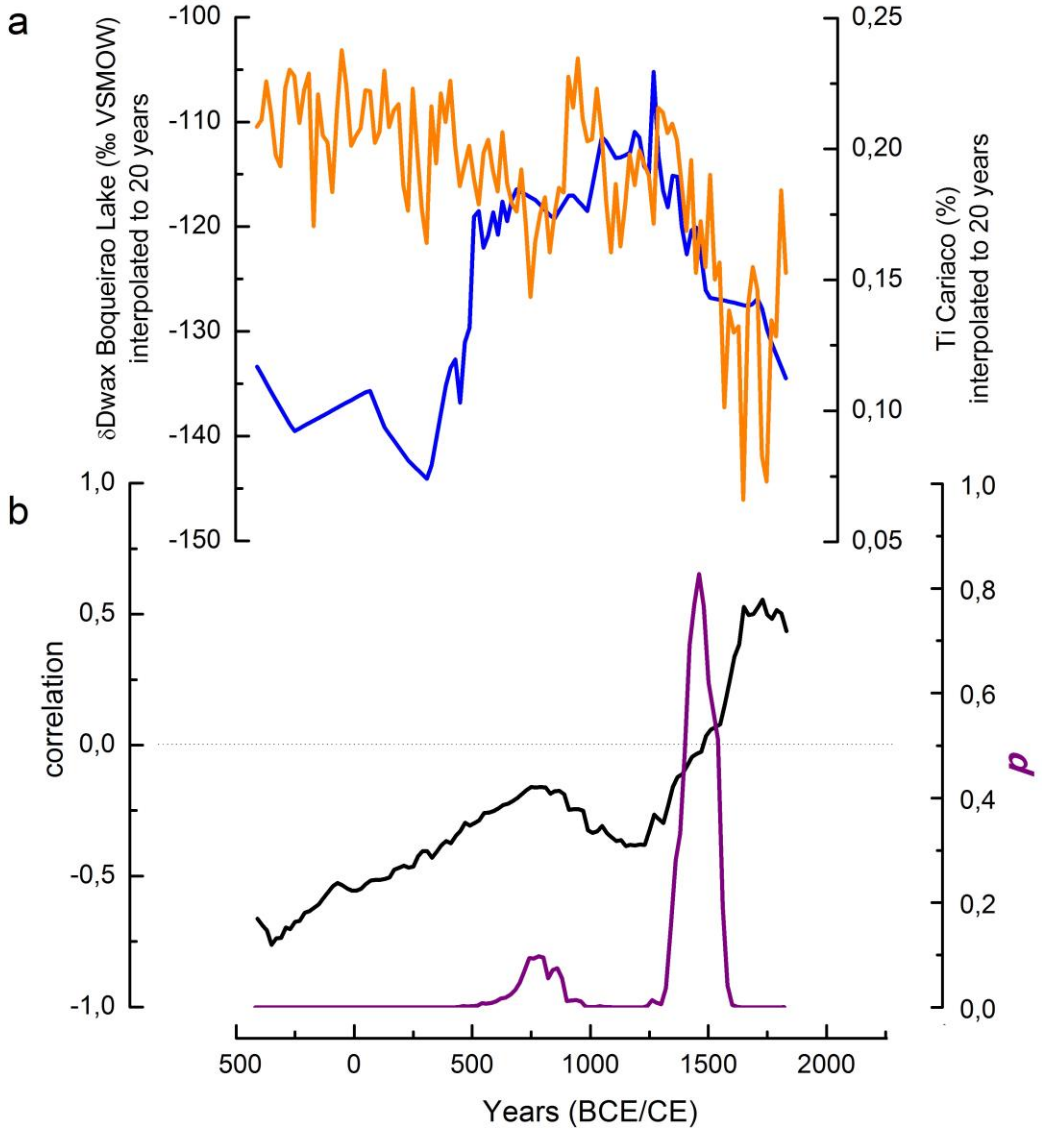


Figure S9 – Running-mean correlation between Boqueirão Lake and Cariaco Basin records[9]. a) Boqueirão Lake $\delta D_{wax}$ and Cariaco Basin Ti concentration[9] interpolated to 20 years. b) Correlation results. The correlation (black line) is negative (positive) when the climate is in-phase (anti-phased). The purple line is the *p* value, only the two peaks have *p* values larger than 0.05. Length of correlation window equals 20 years of interpolated data.

## REFERENCES CITED


1 Schneider, U. et al. GPCC Full Data Reanalysis Version 7.0 at 0.5°: Monthly Land-Surface Precipitation from Rain-Gauges built on GTS-based and Historic Data. doi: 10.5676/DWD_GPCC/FD_M_V7_050. (2015).

2 Atwood, A. R. & Sachs, J.P. Separating ITCZ-and ENSO-related rainfall changes in the Galápagos over the last 3 kyr using D/H ratios of multiple lipid biomarkers. *Earth Planet. Sci.* **404**, 408-419, doi: 1 0.1016/j.epsl.2014.07.038 (2014).

3 Conroy, J.L., Overpeck, J.T., Cole, J.E., Shanahan, T.M. & Stenitz-Kannan, M. Holocene changes in eastern tropical Pacific climate inferred from a Galápagos lake sediment record. *Quat. Sci. Rev.* **27**, 1166–1180, doi: 10.1016/j.quascirev.2008.02.015 (2008).

4 Yamoah, K.A. et al. A 2000-year leaf wax-based hydrogen isotope record from Southeast Asia suggests low frequency ENSO-like teleconnections on a centennial timescale. *Quat. Sci. Rev.* **148**, 44-53, doi: 10.1016/j.quascirev.2016.07.002 (2016).

5 Yan, H. et al. A record of the Southern Oscillation Index for the past 2,000 years from precipitation proxies. *Nat. Geosc*. **4**, 611-614, doi: 10.1038/NGEO1231 (2011).

6 Collister, J.W. et al. Compound-specific $\delta^{13}C$ analyses of leaf lipids from plants with differing carbon dioxide metabolisms. *Org. Geochem*., **21** (**6-7**). 619-627. (1994).

7 Zular, A. *et al*. The effects of mid-Holocene fluvio-eolian interplay and coastal dynamics on the formation of dune-dammed lakes in NE Brazil. *J. Quat. Sci. Rev.* **196**, 137-153, doi: 10.1016/j.quascirev.2018.07.022 (2018).

8 Davis, J.C. 1986. Statistics and data analysis in geology. 638 (John Wiley & Sons, 1986).

9 Haug, G.H. et al. Southward migration of the Intertropical Convergence Zone through the Holocene. *Science*, **293** (5533), 1304-1308, doi: 10.1126/science.1059725 (2001).
